# A Beautiful Case of Instruments

**Published:** 1846-12

**Authors:** 


*A Beautiful Case of Instruments.*—We have just had the pleasure of examining the case of instruments designed as the award by the faculty of the

Baltimore College of Dental Surgery, to the graduating student who shall show the greatest proficiency in the several branches there taught. It is truly a splendid affair, and does the manufacturer, Mr. Francis Arnold, great credit both for taste and skill in his profession. The case is a beautifully mounted rosewood box, elegantly lined with rich velvet and is adapted to every pair of forceps. It contains an entire set of extracting forceps, got up in the most perfect style, and of the patterns invented by Prof. C. A. Harris. The handles of each forcep are ornamented with stones richly set. Had this case of instruments been shown us without being made acquainted with the object, we should have supposed them fitted up with a view of obtaining a premium at a national fair, and that with a good chance of success. The award is to be made by three disinterested persons.—*Syracuse Ed.*

